# Longitudinal Characterization of Coccidiosis Control Methods on Live Performance and Nutrient Utilization in Broilers

**DOI:** 10.3389/fvets.2019.00468

**Published:** 2020-01-14

**Authors:** Alyson E. Gautier, Juan D. Latorre, Phil L. Matsler, Samuel J. Rochell

**Affiliations:** Department of Poultry Science, University of Arkansas, Fayetteville, AR, United States

**Keywords:** broiler, coccidiosis, vaccination, digestibility, growth performance

## Abstract

An experiment was conducted to quantify the timing and magnitude of the effects of coccidiosis vaccination on the growth performance, apparent ileal digestibility (AID) of nutrients and energy, intestinal morphology, and plasma carotenoids and nitric oxide in broilers. Treatment groups consisted of 3 coccidiosis control methods [unvaccinated, unmedicated (NC), in-feed chemical coccidiostat (PC), and live oocyst vaccination (VAC) at day of hatch] administered to male Cobb broilers reared in floor pens. Body weight gain (BWG), feed intake (FI), and feed conversion ratio (FCR) were determined at 12, 16, 20, 28, and 36 d. Blood and ileal digesta were collected from birds in 10 replicate pens of each treatment at 12, 16, 20, and 36 d to evaluate plasma carotenoid and nitric oxide concentrations and determine nutrient AID and IDE. Jejunal samples were taken at 12, 20, and 36 d for morphological measurements. Oocyst shedding in VAC birds was confirmed by increased oocyst counts and decreased carotenoid concentrations (*P* < 0.05) when compared with PC birds, with no differences (*P* > 0.05) in nitric oxide concentrations. At 20 d, BWG and FI were lowest (*P* < 0.05) in VAC birds, intermediate in NC birds, and highest in PC birds, with no differences in FCR (*P* > 0.05). By 28 and 36 d, FCR was higher (*P* < 0.05) for VAC and NC birds but BWG and FI of VAC birds were similar (*P* > 0.05) to PC birds. At d 12, IDE and AID of nitrogen and ether extract were lower (*P* < 0.05) in VAC birds than PC birds. At d 16, AID of nitrogen was similar (*P* > 0.05) between PC and VAC birds, whereas AID of ether extract remained lower in VAC birds than PC birds. No differences in AID of nutrients or IDE were observed (*P* > 0.05) between VAC and PC birds at 20 or 36 d. No differences (*P* > 0.05) in jejunal morphology were observed at any time point. Overall, VAC elicited a transient reduction in AID and IDE, particularly for lipids, that diminished by d 20.

## Introduction

Coccidiosis, an intestinal parasitic disease caused by protozoa of the genus *Eimeria*, remains one of the most prevalent diseases in commercial poultry production. Traditionally, coccidiosis has primarily been managed with in-feed administration of anticoccidial ionophores, but in the United States, ionophores are typically not used in antibiotic-free poultry production systems. Although such systems do currently permit the use of chemical anticoccidial drugs, there is a limited number of these compounds and their overuse can quickly lead to emergence of drug resistant *Eimeria* strains (1, 2). Therefore, this resistance has increased reliance on live oocyst coccidiosis vaccination, which can induce immunity and reintroduce drug-sensitive strains into the rearing facility, as an important control strategy for coccidiosis (3).

Coccidiosis vaccination involves exposure of young chicks, typically at hatch, to small numbers of live *Eimeria* oocysts to promote immunity and reduce the potential for clinical coccidiosis outbreaks during the later growth periods (3). However, mild infections that occur during the process of vaccinal oocyst cycling, which involves the initial infection, oocyst shedding and sporulation, and reinfection, may compromise broiler growth through reduced feed intake or feed efficiency, and the relatively short lifespan of broilers may be insufficient for compensatory gain (4, 5). Impaired feed efficiency during vaccine cycling is presumably due in part to nutrient malabsorption associated with intestinal damage and inflammation associated with the sub-clinical, vaccine-induced infection (5–7).

Reductions in nutrient and energy digestibility have been reported in cocci-exposed birds, and responses are dependent on diet composition and the type and number of *Eimeria* species administered in the challenge model (8–12). Increasing the dietary concentration of digestible nutrients for which digestibility is impaired is a potential strategy to support the performance of broilers during coccidial vaccine cycling. Indeed, Adedokun et al. (7) fed increased concentrations of supplemental amino acids to broilers to account for a predicted reduction in amino acid digestibility based on a previous coccidial challenge trial and observed improved feed efficiency of broilers compared those fed a control diet. However, commercial adoption of this approach for floor-reared, vaccinated broilers requires longitudinal characterization of nutrient digestibility to identify appropriate dietary adjustments that may benefit broilers during the critical stages of vaccination.

Most research to characterize the impact of coccidial challenges on nutrient utilization in broilers has been conducted in battery cages equipped with wire flooring, which prevents multiple infections during oocyst cycling (7–9, 11). Additionally, most of these experiments have involved acute challenges that are much more severe and occur later compared with the mild infections that occur within the first 3 weeks of a vaccinated commercial broiler flock. Therefore, the objective of this study was to characterize the timing and magnitude of reductions in growth performance and nutrient utilization in floor-reared broilers throughout different stages of oocyst cycling following live *Eimeria* vaccination of broilers at day of hatch.

## Materials and Methods

### General Bird Husbandry and Diets

One thousand, five-hundred Cobb 500 male broiler chicks were obtained from a commercial hatchery on day of hatch. All chicks were group-weighed and distributed to 120 floor pens on clean litter with fresh pine shavings. Each floor pen was equipped with a hanging feeder and a nipple drinker line. To ensure sufficient digesta content, 14 birds were placed (0.07 m^2^ per bird) in 30 pens pre-selected for the first collection time point at 12 d post-hatch, whereas 12 birds (0.08 m^2^ per bird) were placed in the other 90 pens to be used for subsequent collections. Birds were provided access to feed and water *ad libitum* throughout the experiment. The lighting schedule and temperature targets were adjusted according to management guidelines published by the primary breeder (13). Birds were reared up to 36 d post-hatch and fed starter (0–14 d), grower (15–28 d), and finisher (29–36 d) diets based on corn and soybean meal and formulated to meet or exceed published nutrient recommendations (13) (Table 1).

**Table 1:** Composition of experimental diets fed to broilers from 0 to 36 d post-hatch^a^.

**Ingredient, % as-fed**	**Starter (0–14 d)**	**Grower (15–28 d)**	**Finisher (29–36 d)**
Corn	57.68	61.11	62.02
Soybean meal (46.8%)	32.90	27.08	23.61
DDGS	4.00	6.00	8.00
Soybean oil	1.34	2.00	2.92
Limestone	1.25	1.22	1.17
Dicalcium phosphate	0.90	0.74	0.52
Salt	0.45	0.42	0.41
DL-methionine	0.31	0.26	0.22
L-lysine HCl	0.24	0.24	0.22
L-threonine	0.09	0.08	0.07
Trace mineral premix^b^	0.10	0.10	0.10
Vitamin premix^c^	0.10	0.10	0.10
Se premix^d^ (0.06%)	0.02	0.02	0.02
Choline chloride (60%)	0.05	0.04	0.04
Santoquin	0.02	0.02	0.02
Phytase^e^	0.01	0.01	0.01
Titanium dioxide	0.50	0.50	0.50
Inert filler^f^	0.05	0.05	0.05
**Calculated composition, % unless noted otherwise**			
AME_n_, kcal/kg	3,015	3,098	3,175
CP	22.01	20.00	19.00
Digestible lysine	1.18	1.05	0.95
Digestible TSAA	0.89	0.80	0.74
Digestible threonine	0.77	0.69	0.65
Calcium	0.90	0.84	0.76
Available P	0.45	0.42	0.38
**Analyzed composition, % unless noted otherwise**			
Gross energy, kcal/kg	3,991	4,027	4,059
CP	22.00	19.60	19.25
Ether extract	4.80	5.57	6.52
Starch	46.89	47.71	56.33

a *DDGS, distillers dried grains with solubles; AME_n_, nitrogen-corrected apparent metabolizable energy*.

b *Supplied the following per kg of diet: manganese, 100 mg; zinc, 100 mg; copper, 10.0 mg; iodine, 1.0 mg; iron, 50 mg; magnesium, 27 mg*.

c *Supplied the following per kg of diet: vitamin A, 30,863 IU; vitamin D3, 22,045 ICU; vitamin E, 220 IU; vitamin B12, 0.05 mg; menadione, 6.0 mg; riboflavin, 26 mg; d-pantothenic acid, 40 mg; thiamine, 6.2 mg; niacin, 154 mg; pyridoxine, 11 mg; folic acid, 3.5 mg; biotin, 0.33 mg*.

d *Supplied 0.12 mg of selenium per kg of diet*.

e *Optiphos®, (Huvepharma Inc., Peachtree City, GA.) provided 250 FTU/kg of diet*.

f *Clinacox®, (Huvepharma Inc., Peachtree City, GA), provided 1 mg/kg diclazuril to the diet at the expense of the inert filler*.

### Experimental Treatments

Upon arrival, one-third (500) of the chicks were orally-gavaged with the manufacturer's recommended dose of a live oocyst vaccine (Coccivac®-B52; Merck Animal Health, Intervet Inc. Millsboro, DE, USA). An oral gavage (0.25 mL/bird) was used to provide uniform administration. Throughout the trial, litter was sprayed once daily with water using a handheld garden sprayer to ensure sufficient moisture content for oocyst sporulation. Unvaccinated broilers were fed diets formulated with or without an in-feed chemical anticoccidial drug, resulting in a total of three treatments: (1) unmedicated and unvaccinated (NC), (2) in-feed chemical coccidiostat (Clinacox, Huvepharma) administration (PC), and (3) live oocyst vaccination (VAC) at day of hatch. Each treatment group was represented by 10 replicate pens for each collection time point.

### Measurement of Live Performance and Vaccine Cycling

Birds and feeders were weighed at 0, 12, 16, 20, 28, and 36 d post-hatch for calculation of body weight gain (BWG), feed intake (FI), and feed conversion ratio (FCR). All dead and culled birds were weighed individually and FCR calculations were adjusted to include the weight gain of dead birds. To assess vaccine cycling, the number of oocysts per gram (OPG) of litter samples collected from each pen was determined before bird placement and at 12, 16, 20, 28, and 36 d post-vaccination. Samples were taken from 3 or 4 different locations within each pen and pooled into airtight plastic bags and kept refrigerated until further analysis. All sample counts were conducted within 1 week of collection. Samples (~150 g) were soaked in water (~1,000 mL) overnight and the solution was vigorously stirred until excreta appeared to be completely solubilized. A 1 ml subsample was further diluted with 9 ml of saturated salt solution and pipetted into the chamber of a McMaster counting slide. Duplicate counts were made for each sample and subsequent calculations based on the following equation:

$$\begin{array}{l} Oocysts\;per\;gram\;of\;sample\\ =\frac{\left(Oocyst\;count\;x\;diluation\;x\;volume\right)}{\left(volume\;of\;counting\;chamber\;x\;weight\;of\;sample\right)} \end{array}$$

where the dilution was 10 and the volume of the counting chamber was 0.15 ml.

### Digesta, Blood, and Tissue Sampling

To avoid changes in stocking density among time points due to bird sampling, all birds from 10 replicate pens of each treatment were humanely euthanized at 12, 16, 20, and 36 d post-hatch by CO_2_ inhalation for collection of ileal digesta. Ileal contents from all birds in each pen were collected by gently flushing the distal half of the ileum using deionized water. Digesta samples were pooled within pen and frozen (−20°C) until analysis. At 12, 16, and 36 d post-hatch, 2 birds from the same pens used for digesta collection were randomly selected for blood and jejunal tissue collection and pH determination of duodenal lumen contents. Blood was collected immediately post-mortem via cardiac puncture into tubes containing EDTA, placed on ice, and centrifuged for 15 min at 1,300 × *g* and 4°C to separate plasma. Plasma from birds within a pen were pooled, aliquoted, and stored at −80°C until further analysis. To determine duodenal pH, a digital pH meter (Mettler-Toledo, UK) with a spear tip piercing pH electrode (Sensorex S175CD) was directly inserted into the digesta in the distal duodenal loop and the pH was recorded. The probe was rinsed with distilled water after each reading and the tip of the pH probe was stored in pH 4 solution when not in use. Jejunal tissue samples (~2 cm in length) were collected at the midpoint of the jejunum between the end of the duodenal loop and the Meckel's diverticulum and rinsed with PBS to remove luminal contents and placed in scintillation vials containing 10% neutral-buffered formalin.

### Laboratory Analyses

Frozen digesta samples were lyophilized and ground using an electric coffee grinder to provide an evenly ground sample while avoiding significant loss. Diet and digesta samples were analyzed for dry matter, gross energy, nitrogen, ether extract, and starch content. Dry matter was determined according to AOAC (14) method 934.02. Gross energy was determined with a bomb calorimeter (Parr 6200 bomb calorimeter, Parr Instruments Co., Moline, IL.). Nitrogen was determined using the combustion method (Fisions NA-2000, CE Elantech, Lakewood, NJ) standardized with EDTA [method 990.03, (14)] and ether extract was determined according to AOAC (14) method 920.39. Starch concentrations of feed and digesta samples were measured using the Megazyme Total Starch Assay Kit according to instructions provided by the manufacturer (Megazyme Int. Ireland Ltd., Wicklow, Ireland). Titanium dioxide was included in the feed at 0.5% as an indigestible marker, and diet and digesta TiO_2_ concentrations were determined in duplicate following the procedures of Short et al. (15). Apparent ileal digestibility (AID) of dry matter, gross energy, ether extract, nitrogen, and starch were calculated using the following equation:

$$AID,\%=\left\{\frac{\left[(\frac{\text{X}}{\text{TiO}2}\right)\text{diet}-\left(\frac{\text{X}}{\text{TiO}2}\right)\text{digesta}]}{\left(\frac{\text{X}}{\text{TiO}2}\right)\text{diet}}\right\}\times 100$$

where (X/TiO_2_) = ratio of nutrient concentration to TiO_2_ in the diet or ileal digesta. Energy digestibility (%) values obtained from the equation above were multiplied by the gross energy content of the feed to calculate ileal digestible energy (IDE).

Plasma samples were analyzed to determine carotenoid and nitric oxide concentrations. All blood processing and carotenoid analysis procedures were conducted under yellow light. Plasma carotenoid concentrations were determined by spectrophotometry as previously described by Allen (16). Plasma nitrate (NO3–) + nitrite (NO2–) concentrations were measured to determine total nitric oxide using a colorimetric assay kit (Cayman Chemical CO., Ann Arbor, MI). Prior to nitric oxide analysis, plasma samples were filtered through pre-rinsed centrifugal filters (VWR, Radnor, PA) to remove potentially interfering proteins with a molecular weight >30 kDa.

Jejunal tissue samples were embedded in paraffin, sectioned at 4 μm, set on a glass slide, and stained with hematoxylin and eosin. Photomicrographs of each jejunum sample were acquired using a light microscope (Nikon Eclipse) equipped with a digital camera. Imaging software (Nikon's NIS Elements Basic Research Microscope Imaging) was used for measurement of villus height, crypt depth, and villus width under 4x magnification. For villus height, ~6 intact well-oriented villi per bird were randomly selected and measured. Villus height was measured from the tip of the villus to the villus-crypt junction, whereas crypt depth was defined as the depth of the invagination between adjacent villi. The width of the villus was measured at the basal (crypt-villus junction) and apical ends (17). Apparent jejunal villus surface area was calculated using the following equation published by Iji et al. (17):

$$\begin{array}{l} Apparent\;villus\;surface\;area\\ =\frac{\text{((}villus\;basal\;width\;+\;villus\;apical\;width\text{)}}{\left(2\;x\;villus\;height)\right)} \end{array}$$

### Statistical Analysis

Pen was considered the experimental unit with 10 replicate pens per treatment for each collection time point. Treatment groups were arranged in randomized complete block design and the statistical model included pen location as the random blocking factor. Data within each time point were subjected to ANOVA using the MIXED procedure of SAS 9.4 and are presented as least squares means of treatment groups. Jejunal morphology data were transformed (log 10) to meet normality assumptions for the ANOVA. Statistically different treatment means were separated using a Tukey's multiple comparison test. Statistical significance was considered at *P* < 0.05.

## Results

### Oocyst Shedding and Plasma Measurements

**Figure 1:**
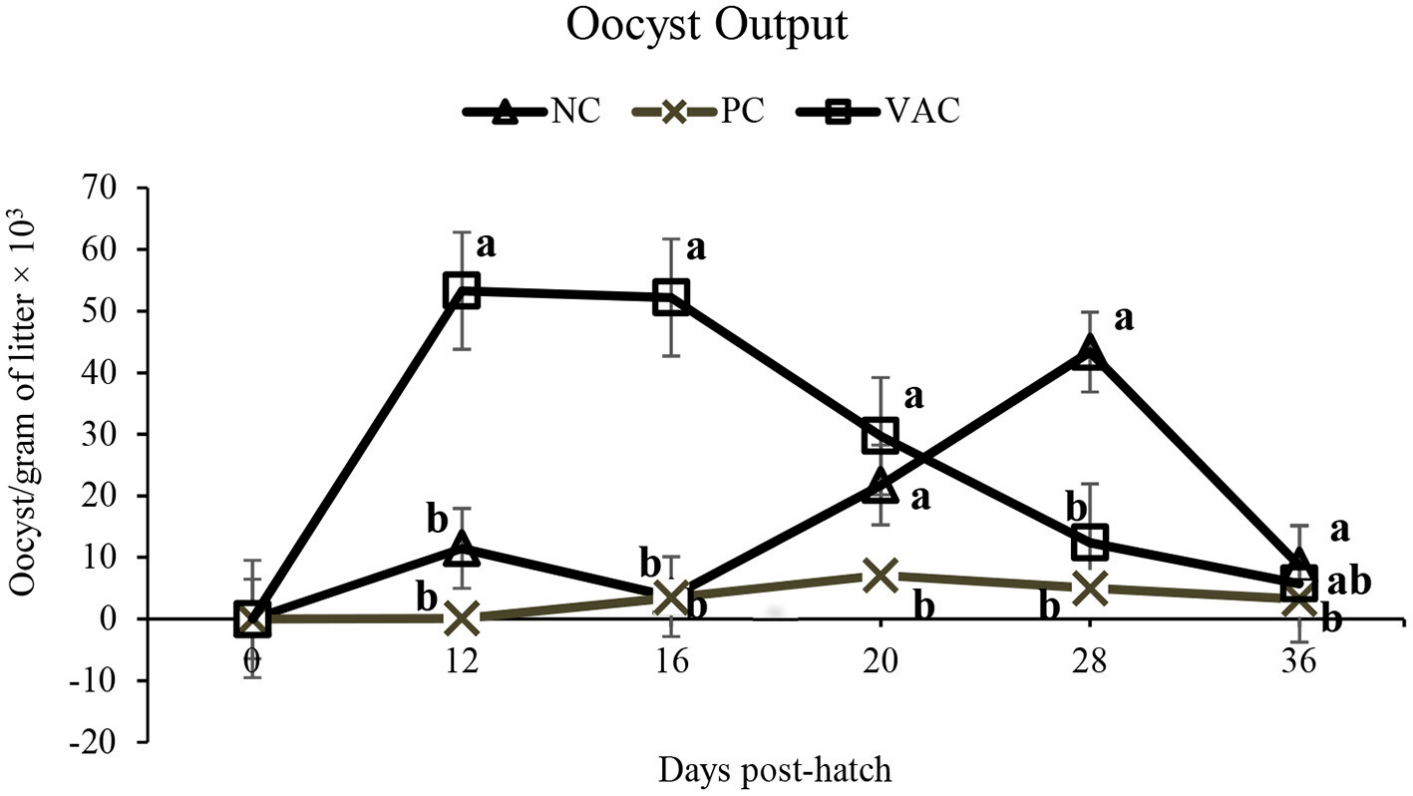
The effects of coccidiosis vaccination on litter oocyst counts from 0 to 36 d post-hatch. Values are LSMeans of 10 replicate pens. NC, negative control; PC, positive control, birds were given an in-feed anticoccidial drug; VAC, vaccinated, birds were given a commercial dose of vaccine on 0 d. Within each time point, lines that do not share a common superscript are different (*P* < 0.05).

Litter oocyst counts of PC birds remained low throughout the experiment (Figure 1). At 12 and 16 d post hatch, OPG counts from VAC birds were significantly (*P* < 0.05) higher than those of NC and PC treatments. At 20 d post-hatch, OPG counts in both NC and VAC birds were higher (*P* < 0.05) than those of PC birds, indicating that NC birds had become inadvertently infected. At 28 d post-hatch, OPG of litter in NC birds was significantly higher (*P* < 0.05) than both PC and VAC treatments, with no differences (*P* > 0.05) observed between PC and VAC birds. At 36 d post-hatch, OPG of litter was highest (*P* < 0.05) in NC birds, intermediate in VAC birds, and lowest in PC birds.

Plasma carotenoid concentrations and plasma nitric oxide levels are presented in Table 2. Plasma carotenoid concentrations in NC and PC birds were higher (*P* < 0.05) than VAC birds at both 12 and 16 d post-hatch. At 20 d post-hatch, plasma carotenoid concentrations for NC and VAC birds were lower (*P* < 0.05) than those of PC birds. At 36 d, no differences (*P* > 0.05) in plasma carotenoids were observed among the treatments. Plasma nitric oxide concentrations were unaffected (*P* > 0.05) by any of the treatments, regardless of collection time point.

**Table 2:** Effects of coccidiosis vaccination on plasma carotenoid and nitric oxide concentrations from 12 to 36 d post-hatch^1^^,^^2^.

**Item**	**NC**	**PC**	**VAC**	**SEM**	***P*-value**
**12 d**					
Plasma carotenoids, μg/mL	2.28^a^	2.38^a^	1.15^b^	0.174	0.001
Nitric oxide, μM	8.41	8.40	8.18	0.483	0.933
**16 d**					
Plasma carotenoids, μg/mL	2.05^a^	2.21^a^	1.30^b^	0.194	0.006
Nitric oxide, μM	9.13	8.75	8.19	0.481	0.399
**20 d**					
Plasma carotenoids, μg/mL	0.54^b^	1.29^a^	0.48^b^	0.134	0.001
Nitric oxide, μM	6.03	7.13	7.06	0.418	0.133
**36 d**					
Plasma carotenoids, μg/mL	3.37	4.24	3.14	0.315	0.050
Nitric oxide, μM	8.26	6.99	8.24	0.630	0.279

a,b *Means within a row that do not share a common superscript are different (P < 0.05)*.

1 *Values are LSMeans of 10 replicate pens*.

2 *NC, negative control; PC, positive control, birds were given an in-feed anticoccidial drug; VAC, birds were given a commercial dose of vaccine on 0 d*.

### Growth Performance

No differences (*P* > 0.05) in BWG, FI, or FCR were observed among birds in any of the treatment groups from 0 to 12 d or 0 to 16 d post-hatch (Table 3). From 0 to 20 d post-hatch, BWG and FI were lowest (*P* < 0.05) in VAC birds, intermediate in NC birds, and highest in PC birds, with no differences (*P* > 0.05) in FCR. By 28 d post-hatch, BWG in VAC birds did not differ (*P* > 0.05) from PC and NC birds, whereas NC birds had a lower (*P* < 0.05) BWG when compared with PC birds. No differences (*P* > 0.05) in FI were observed among treatments at 28 d post-hatch; however, NC and VAC birds had a higher (*P* < 0.05) FCR when compared with PC birds. By 36 d post-hatch, FCR remained higher (*P* < 0.05) in NC and VAC compared with PC birds, whereas BWG of broilers and FI were not influenced (*P* > 0.05) by treatment.

**Table 3:** Effects of coccidiosis vaccination on growth performance of broilers from 0 to 36 d post-hatch^1^^,^^2^.

**Item**	**NC**	**PC**	**VAC**	**SEM**	***P*-value**
**0–12 d**					
d12 BW, kg/bird	0.355	0.350	0.347	0.004	0.362
BW gain, kg/bird	0.312	0.308	0.305	0.004	0.414
Feed intake, kg/bird	0.400	0.397	0.397	0.005	0.880
FCR	1.279	1.292	1.305	0.010	0.183
**0–16 d**					
d16 BW, kg/bird	0.544	0.548	0.531	0.006	0.133
BW gain, kg/bird	0.502	0.505	0.489	0.006	0.115
Feed intake, kg/bird	0.736	0.742	0.724	0.007	0.185
FCR	1.468	1.473	1.488	0.011	0.440
**0–20 d**					
d20 BW, kg/bird	0.892^a^^b^	0.909^a^	0.877^b^	0.008	0.038
BW gain, kg/bird	0.849^a^^b^	0.867^a^	0.834^b^	0.008	0.036
Feed intake, kg/bird	1.138^a^^b^	1.164^a^	1.117^b^	0.009	0.006
FCR	1.341	1.345	1.341	0.011	0.961
**0–28 d**					
d28, kg/bird	1.602^b^	1.682^a^	1.647^a^^b^	0.016	0.007
BW gain, kg/bird	1.560^b^	1.639^a^	1.605^a^^b^	0.016	0.007
Feed intake, kg/bird	2.334	2.375	2.393	0.019	0.096
FCR	1.501^a^	1.454^b^	1.493^a^	0.007	0.001
**0–36 d**					
d36 BW, kg/bird	2.512	2.588	2.555	0.024	0.086
BW gain, kg/bird	2.469	2.545	2.513	0.024	0.087
Feed intake, kg/bird	3.882	3.905	3.950	0.027	0.191
FCR	1.578^a^	1.543^b^	1.576^a^	0.009	0.018

a,b *Means within a row that do not share a common superscript are different (P < 0.05)*.

1 *Values are LSMeans of 9 or 10 replicate pens*.

2 *NC, negative control; PC, positive control, birds were given an in-feed anticoccidial drug; VAC, vaccinated, birds were given a commercial dose of vaccine on 0 d*.

### Apparent Ileal Digestibility of Nutrients and IDE

Vaccinated birds had lower (*P* < 0.05) IDE and AID of nitrogen, ether extract, and starch compared with NC and PC birds at 12 d post-hatch (Table 4). By 16 d post-hatch, no differences (*P* > 0.05) in AID of nitrogen, starch, or IDE were observed among treatment groups, but AID of ether extract remained lower (*P* < 0.05) in VAC birds than in NC or PC birds. At 20 d post-hatch, no differences (*P* > 0.05) in AID or IDE were observed between PC and VAC birds, however, NC birds had lower (*P* < 0.05) AID of nitrogen, ether extract, and IDE compared with PC and VAC birds. By 36 d post-hatch, no differences (*P* > 0.05) in IDE and AID of nitrogen or ether extract were observed among treatment groups. At 36 d, AID of starch remained lower (*P* < 0.05) in NC birds compared with PC birds, with no difference (*P* > 0.05) in AID of starch between PC and VAC birds.

**Table 4:** Effects of coccidiosis vaccination on the apparent ileal digestibility (%) of nutrients and ileal digestible energy (kcal/kg) in broilers to 36 d post-hatch^1^^,^^2^.

**Item**	**NC**	**PC**	**VAC**	**SEM**	***P*-value**
**12 d**					
Dry matter, %	70.4^a^	71.5^a^	66.9^b^	0.58	0.001
Nitrogen, %	81.7^a^	82.8^a^	77.6^b^	0.55	0.001
Ether extract, %	88.5^a^	89.3^a^	79.6^b^	1.72	0.001
Starch, %	90.7^a^	92.0^a^	88.9^b^	0.41	0.001
IDE, kcal/kg^3^	3,337^a^	3,399^a^	3,138^b^	26	0.001
**16 d**					
Dry matter, %	74.9	73.3	74.0	0.52	0.086
Nitrogen, %	83.9	83.4	83.4	0.45	0.706
Ether extract, %	91.1^a^	91.0^a^	85.9^b^	1.38	0.017
Starch, %	91.0	89.7	89.7	0.76	0.380
IDE, kcal/kg^3^	3,587	3,542	3,543	24	0.317
**20 d**					
Dry matter, %	71.1^b^	72.6^a^	73.2^a^	0.43	0.005
Nitrogen, %	79.6^b^	82.4^a^	81.7^a^	0.46	0.004
Ether extract, %	65.1^b^	85.9^a^	78.6^a^	3.53	0.001
Starch, %	90.4^b^	91.7^a^	91.3^a^^b^	0.34	0.028
IDE, kcal/kg^3^	3,326^b^	3,469^a^	3,447^a^	25	0.001
**36 d**					
Dry matter, %	75.5^a^	75.6^a^	73.7^b^	0.41	0.005
Nitrogen, %	83.5	83.1	83.7	0.41	0.398
Ether extract, %	94.0	94.1	92.5	0.88	0.271
Starch, %	89.0^b^	90.9^a^	89.4^a^^b^	0.48	0.023
IDE, kcal/kg^3^	3,629	3,642	3,605	19	0.404

a,b *Means within a row that do not share a common superscript are different (P < 0.05)*.

1 *Values are LSMeans of 10 replicate pens*.

2 *NC, negative control; PC, positive control, birds were given an in-feed anticoccidial drug; VAC, vaccinated, birds were given a commercial dose of vaccine on 0 d*.

3 *IDE, ileal digestible energy*.

### Jejunal Morphology and Duodenal pH

No significant differences (*P* > 0.05) in intestinal jejunal morphology or duodenal pH were observed among any of the treatment groups at any time point (Table 5). However, at 12 d post-hatch, there was a tendency (*P* = 0.07) for VAC birds to have deeper jejunal crypts than NC and PC birds. Furthermore, at 36 d post-hatch, there was a tendency (*P* = 0.07) for VAC birds to have reduced jejunal villus heights compared with NC and PC birds.

**Table 5:** Effects of coccidiosis vaccination on jejunal morphology and duodenal pH of broilers in 36 d post-hatch^1^^,^^2^.

**Item**	**NC**	**PC**	**VAC**	**SEM**	***P*-value^3^**
**12 d**					
Villus height, μm	645 (2.81)	669 (2.82)	693 (2.84)	23.0 (0.015)	0.392
Crypt depth, μm	124 (2.08)	118 (2.82)	145 (2.84)	5.7 (0.028)	0.082
Villus height to crypt depth	5.81 (0.75)	6.60 (0.81)	5.38 (0.71)	0.372 (0.037)	0.159
Villus surface area, mm^2^	0.10 (5.01)	0.10 (5.01)	0.10 (4.98)	0.006 (0.025)	0.772
pH	5.64	5.61	5.57	0.074	0.773
**20 d**					
Villus height, μm	774 (2.89)	730 (2.86)	786 (2.90)	24.1 (0.014)	0.189
Crypt depth, μm	200 (2.28)	188 (2.26)	211 (2.31)	13.4 (0.029)	0.561
Villus height to crypt depth	4.58 (0.64)	4.40 (0.62)	4.36 (0.62)	0.352 (0.033)	0.891
Villus surface area, mm^2^	0.13 (5.10)	0.13 (5.11)	0.13 (5.12)	0.006 (0.021)	0.800
pH	6.11	6.08	6.12	0.028	0.628
**36 d**					
Villus height, μm	1,058 (3.02)	1,100 (3.04)	1,005 (3.01)	27.7 (0.012)	0.071
Crypt depth, μm	177 (2.23)	153 (2.17)	169 (2.21)	14.4 (0.035)	0.507
Villus height to crypt depth	7.10 (0.83)	8.14 (0.90)	6.96 (0.82)	0.672 (0.040)	0.306
Villus surface area, mm^2^	0.19 (5.28)	0.20 (5.30)	0.19 (5.26)	0.001 (0.029)	0.653
pH	6.12	6.15	6.12	0.015	0.166

1 *Values are LSMeans of 10 replicate pens with transformed data (log_10_) used for statistical analysis in parentheses*.

2 *NC, negative control; PC, positive control, birds were given an in-feed anticoccidial drug; VAC, vaccinated, birds were given a commercial dose of vaccine on 0 d*.

3 *P-values represent transformed data (log_10_)*.

## Discussion

Coccidiosis vaccines can prevent coccidiosis outbreaks in broiler flocks but can induce damage to the intestinal epithelium, impair live performance, and potentially increase susceptibility to other enteric diseases such as necrotic enteritis. The objective of this experiment was to characterize the timing and magnitude by which coccidiosis vaccination at day of hatch influences growth performance and nutrient utilization in floor-reared broilers during the various stages of *Eimeria* cycling. Litter oocyst counts and plasma carotenoids indicated that in-feed diclazuril administration prevented coccidial infection in PC birds, whereas increased oocyst shedding and decreased plasma carotenoid concentrations reflected cycling of vaccinal oocysts in VAC broilers. This finding aligned with previous reports that plasma carotenoids are a sensitive indicator of coccidial-induced intestinal damage in chickens (18–20) and confirmed that the most commercially-important comparison of the medicated PC group and VAC remained valid throughout the experiment. However, these same measurements indicated an inadvertent infection of the NC group, which likely occurred due to the fact that all treatment groups were distributed evenly in blocks throughout a single experimental facility to minimize environmental or location-related effects. Although the infection of the NC group was unintended, this did provide another time point at which to compare the impact of infection on the responses measured which, as described below, generally aligned with the responses observed in the VAC group at 12 and 16 d post-hatch.

Coccidiosis vaccines can induce coccidiasis, a mild transient form of coccidiosis, usually occurring between 14 and 28 d post-hatch, which can impair broiler performance (5). Coccidiosis vaccination at day of hatch did not impact broiler performance at 12 or 16 d post-hatch in the current experiment, but it did reduce FI and BWG of broilers at 20 d post-hatch, with no effects on FCR. The reductions in BWG and FI for VAC birds, compared with PC birds, had diminished by 28 d. Lehman et al. (5) also reported vaccinated broilers had a reduction in BWG at 21 d of age, although this reduction was a result of impaired FCR and not reduced FI. Furthermore, Silva et al. (21) similarly reported a vaccine-induced reduction in BWG at 21 d of age but observed no differences in BWG between vaccinated and non-vaccinated birds at 36 d of age. Indeed, the goal of coccidiosis vaccination is to provide an early *Eimeria* exposure to allow sufficient time for the birds to compensate for the minor reduction in weight before the end of the grow-out period (22). The decreased BWG in NC birds relative to PC birds at 28 d post-hatch is in agreement with the litter oocyst counts and further reflects an inadvertent infection on NC birds at this time. However, while VAC and PC birds did not differ in BWG or FI at 28 d, FCR at 28 and 36 d post-hatch remained higher for NC and VAC birds than for PC birds, possibly due to nutrient malabsorption throughout the experiment.

The greatest impacts of vaccination on nutrient and energy digestibility were observed at 12 d post-hatch, which likely corresponds with the second *Eimeria* life cycle (23). Specifically, vaccination decreased IDE by 261 kcal/kg and AID of nitrogen, ether extract, and starch by 5.2, 9.7, and 3.1 percentage units, respectively (6.3, 10.9, and 3.4 percentage reduction, respectively) compared with PC birds at 12 d post-hatch. Reduced nitrogen digestibility leads to an increased amount of protein in the terminal ileum, which can be fermented to produce toxic compounds such as biogenic amines (24, 25). While differences in nitrogen digestibility between VAC and PC birds had diminished by 16 d post-hatch, the impaired 12 d digestibility may onset early intestinal bacteria overgrowth to predispose the birds to secondary infections, such as necrotic enteritis, that typically manifests between the second and fifth week of age (26).

Caloric costs due to coccidiosis vaccination at 12 d post-hatch, as reflected by reduced IDE, were associated with reductions in ether extract and starch digestibility. However, no differences in starch or IDE were observed between VAC and PC birds by 16 d post-hatch. On the other hand, ether extract digestibility remained 5.1 percentage units lower (6% reduction) in VAC birds than in PC birds at 16 post-hatch. Moreover, the severe impact of a coccidial challenge on lipid digestibility was also observed with the inadvertent infection of NC birds at 20 d post-hatch, whereby digestibility of ether extract was 20.8 percentage units lower (24% reduction) in NC birds compared to PC birds. The variation in ether extract digestibility was also higher at 20 d post-hatch than at earlier time points, and as such, the numerical reduction in ether extract digestibility of 7.3 percentage units (8% reduction) in VAC birds compared with PC birds was not statistically different. The relative impacts on starch and lipid digestibility observed in the current experiment are in agreement with findings by Amerah and Ravindran (10), who reported broilers subjected to a mixed species challenge had an 18.8% reduction in starch and 96% reduction in lipid digestibility at 7 d post-challenge compared with non-challenged broilers. Starch provides a greater relative contribution to the overall energy content of the diet, but has a lower caloric value than that of lipids. Therefore, the impact of overall energy utilization on starch and lipid was determined by multiplying the FI per bird by the analyzed concentration of either starch or lipid, multiplied by the assumed caloric value, either 4 or 9.5 kcal/kg for starch and lipid, respectively. As such, the VAC-induced reductions in starch and lipid digestibility resulted in a similar caloric cost.

Carotenoids are fat-soluble components (27), and the marked reductions in plasma carotenoids observed in the current experiment were likely associated with the observed reductions in lipid digestibility. The profound effect of coccidia on lipid digestibility can subsequently impair the absorption of other fat-soluble nutrients, including vitamin D, which can consequently impair the absorption of calcium and phosphorus, negatively impacting bone development (28–30). Increased digesta lipid content may also reduce the absorption of calcium via intestinal soap formation, and excess calcium in the intestinal lumen may be another predisposing factor for necrotic enteritis (31). However, since lipid digestion and absorption are relatively complex processes, it is currently unknown which of these processes are most impacted during coccidia-exposure. Sharma and Fernando (32) observed an accumulation of lipid globules within the duodenal villus epithelial cells of *E. acervulina* infected birds, indicating that intracellular lipid processing or transport across the basolateral cell membrane of the enterocyte may be compromised. Furthermore, Adams et al. (33) reported coccidiosis challenged birds had an improvement in lipid digestion when supplemented with cholic acid, indicating that bile salt synthesis or secretion may be impaired during an infection.

In the current experiment, jejunum villi height was not influenced by coccidiosis vaccination at 12 d post-hatch when the greatest impacts of vaccination on nutrient and energy digestibility were observed. Although severe coccidiosis can cause morphologic damage to the intestinal mucosa, as indicated by increased crypt cell depth and shortened villi (30, 34), other authors have similarly reported a lack of effects of coccidiosis vaccines on intestinal morphology when administered at commercially recommended doses (35, 36). There was a tendency for VAC birds to have deeper crypts in the jejunum at 12 d post-hatch in the current experiment, and this may be reflective of increased cellular proliferation to maintain villi structure during periods of increased enterocyte turnover associated with vaccine-induced coccidiasis. Indeed, Luquetti et al. (36) reported that coccidiosis vaccination did not affect duodenum, jejunum, or ileum villi heights but did increase jejunum and ileum crypt depths of broilers at 14 d post-hatch. Although increased cellular turnover appears to be sufficient to maintain villus structure under these conditions, it also likely increases intestinal maintenance costs for nutrients and energy (34, 37). Furthermore, rapid enterocyte turnover may reduce nutrient transporter expression and brush border enzyme activity, which would consequently reduce the digestion and absorption of nutrients (38).

It has been suggested that *Eimeria*-induced pH reductions can cause intestinal pH to fall below the optima efficiency for digestive enzyme activity (39). To our knowledge, no published work had evaluated pH in a model that mimics field relevant conditions of coccidiosis vaccination. However, no differences in pH of the duodenum, where pancreatic enzymes are secreted and the majority of enzymatic digestion occurs, were observed in the current experiment. Therefore, the lack of differences observed in histology or pH in coccidiosis vaccinated broilers suggests that these factors alone are not primary contributors to the observed reductions in nutrient digestibility.

Intestinal inflammation may also contribute to the transient reductions in nutrient digestibility experienced by coccidiosis-vaccinated broilers. Nitric oxide is produced by macrophages during the inflammatory response to *Eimeria* infection via the enzyme nitric oxide synthase, and Allen (40) reported that an *E. maxima* infection induced nitric oxide production in the mucosa of the infected intestinal area, as well as in the blood. Recently, Rochell et al. (41) reported that a 40% reduction in dietary arginine, the key substrate for nitric oxide, did not limit the marked increase in plasma nitric oxide elicited by *E. acervulina* infection, indicating a high prioritization of arginine for nitric oxide synthesis during a coccidial challenge. In the current experiment, coccidiosis vaccination did not increase nitric oxide in the plasma, which is in agreement with the findings of Perez-Carbajal et al. (42). Local nitric oxide production in the mucosa was not measured in the current experiment. Nonetheless, it appears that the transient coccidiosis elicited by vaccination does not induce nitric oxide production in the periphery as do more severe challenges.

In conclusion, results reported herein indicate that coccidiosis vaccination had no significant impact on overall BWG and FI of VAC birds, although overall FCR was impaired by vaccination. Coccidiosis vaccination elicited a transient reduction in digestibility of energy and nutrients that was most apparent at 12 post-hatch, particularly for lipids, but VAC birds were able to recover from these reductions by 20 d post-hatch. These results indicate that impaired nutrient digestibility during coccidiosis vaccination may be attributed to a combination of effects, since jejunal morphology and duodenal pH were both not drastically impacted. Furthermore, the prolonged reduction of lipid digestibility in VAC broilers suggests VAC birds have an impaired ability to utilize dietary lipids throughout the various stages of vaccinal oocyst cycling. Therefore, further research is needed to determine the effects of undigested lipids on broiler gastrointestinal health, as well as practical nutrition strategies to ameliorate these effects.

## Data Availability Statement

All datasets generated for this study are included in the article/supplementary material.

## Ethics Statement

The animal study was reviewed and approved by University of Arkansas.

## Author Contributions

SR and JL: designed the experiment. AG: performed the experiment, data analysis, and wrote the paper. AG and PM: laboratory analysis. AG and SR: paper revisions and final approval.

### Conflict of Interest

The authors declare that the research was conducted in the absence of any commercial or financial relationships that could be construed as a potential conflict of interest.
